# Optimal specimen collection and transport methods for the detection of avian influenza virus and Newcastle disease virus

**DOI:** 10.1186/1746-6148-9-35

**Published:** 2013-02-22

**Authors:** Erica Spackman, Janice C Pedersen, Enid T McKinley, Jack Gelb

**Affiliations:** 1 Southeast Poultry Research Laboratory, USDA, Agricultural Research Service, 934 College, Station Road, Athens, GA, 30605, USA; 2 National Veterinary Services Laboratories, USDA-APHIS, Ames, IA, USA; 3 Department of Animal and Food Sciences, University of Delaware, Newark, DE, 19716, USA

## Abstract

**Background:**

Active and passive surveillance for avian influenza virus (AIV) and Newcastle disease virus (NDV) is widespread in commercial poultry worldwide, therefore optimization of sample collection and transport would be valuable to achieve the best sensitivity and specificity possible, and to develop the most accurate and efficient testing programs. A H7N2 low pathogenicity (LP) AIV strain was selected and used as an indicator virus because it is present in lower concentrations in swabbings and thus requires greater sensitivity for detection compared to highly pathogenic (HP) AIV. For similar reasons a mesogenic strain of NDV was selected. Using oro-pharyngeal and cloacal swabs collected from chickens experimentally exposed to the viruses we evaluated the effects of numerous aspects of sample collection and transport: 1) swab construction material (flocked nylon, non-flocked Dacron, or urethane foam), 2) transport media (brain heart infusion broth [BHI] or phosphate buffered saline [PBS]), 3) media volume (2 ml or 3.5 ml), 4) transporting the swab wet in the vial or removing the swab prior to transport, or transporting the swab dry with no media, and 5) single swabs versus pooling 5 or 11 swabs per vial.

**Results:**

Using real-time RT-PCR (rRT-PCR), virus isolation (VI) and commercial antigen detection immunoassays for AIV we observed statistically significant differences and consistent trends with some elements of sample collection and transport; media, dry transport and swab construction. Conversely, the number of swabs pooled (1, 5 or 11) and whether the swab was removed prior to transport did not impact virus detection. Similarly, with NDV detection by both VI and rRT-PCR was not affected by the numbers of swabs collected in a single vial (1, 5 or 11).

**Conclusions:**

We observed that flocked and foam swabs were superior to non-flocked swabs, BHI media was better than PBS, and transporting swabs wet was better for virus recovery and detection than transporting them dry. There was no observable difference in detection whether the swab was removed prior to transport or left in the vial. Also, with both AIV and NDV, there was no observed difference in virus detection between pools of 1, 5 or 11 swabs.

## Background

Surveillance of poultry for notifiable avian influenza virus (AIV) and virulent Newcastle disease virus (NDV) is critical for maintaining export markets under the guidelines of the World Organization for Animal Health (OIE). Moreover, in the US, AIV surveillance is a key component of the US National Poultry Improvement Plan (NPIP). In addition to the extensive level of testing of commercial poultry globally, AIV surveillance is conducted in live-bird markets and in wild birds. Accurate test results, which are affected by collection and transport conditions, are critical for screening since both false negative and false positive results can have negative impacts on disease control and trade.

Surveillance testing for AIV and NDV have been established and successfully implemented in many countries, however there has been minimal work to validate sample collection methods. Evaluations of swab pooling for AIV using only real-time (rRT-PCR), but not virus isolation have been reported [1,2]. Similarly, detection of AIV with wet versus dry swabs from Pekin ducks tested by rRT-PCR only has been compared [3].

Currently, collection methods are not standardized; there are variations depending on poultry species, industry compartment, surveillance objectives and guidelines, and the availability and cost of materials. Therefore questions continually arise from personnel involved in AIV and NDV testing and the design of surveillance programs about what approach is best. Importantly, the specific details of sample collection practices have not been fully evaluated for their effects on the sensitivity and specificity of rRT-PCR or commercial antigen immunoassays (AgIA), and in some cases virus isolation (VI), the most widely used detection tests for AIV. With the large scale of testing currently being conducted, small differences in sensitivity and specificity could translate into a substantial impact on diagnostic accuracy. Therefore, the objective of this work was to determine the optimal sample collection methods for AIV detection based on practical variations in several aspects of AIV sample collection and transport. In addition, pooling of specimens was evaluated for NDV since similar to AIV, both rRT-PCR and VI are used for the detection and surveillance for NDV.

The elements of AIV and NDV sample collection and transport that were evaluated are: 1) swab construction type (material used to make the swab), 2) transport media, 3) transport of dry swabs versus wet swabs, 4) leaving swabs in the media after collection or removing them, 5) media volume, and 6) swab pooling. The conditions selected for evaluation are minor modifications of those currently in use or are new technologies which have shown improvements for public health testing for influenza and other diseases, for example: swab construction, transport conditions and pooling [4-11]. Importantly all elements are expected to be economically and logistically feasible for poultry applications.

## Results

### General

In general, with AIV too few cloacal (CL) swabs were positive by either VI or rRT-PCR for meaningful analysis, therefore the results will focus on oro-pharyngeal (OP) swabs, which are the preferred sample for AIV and NDV detection in gallinaceous poultry. Also no samples were positive at day 10 post inoculation (PI) or later for any experiment.

### Swab construction material and transport media

Since swab construction material type and transport media were evaluated in the same experiment the results will be presented together.

**Figure 1:**
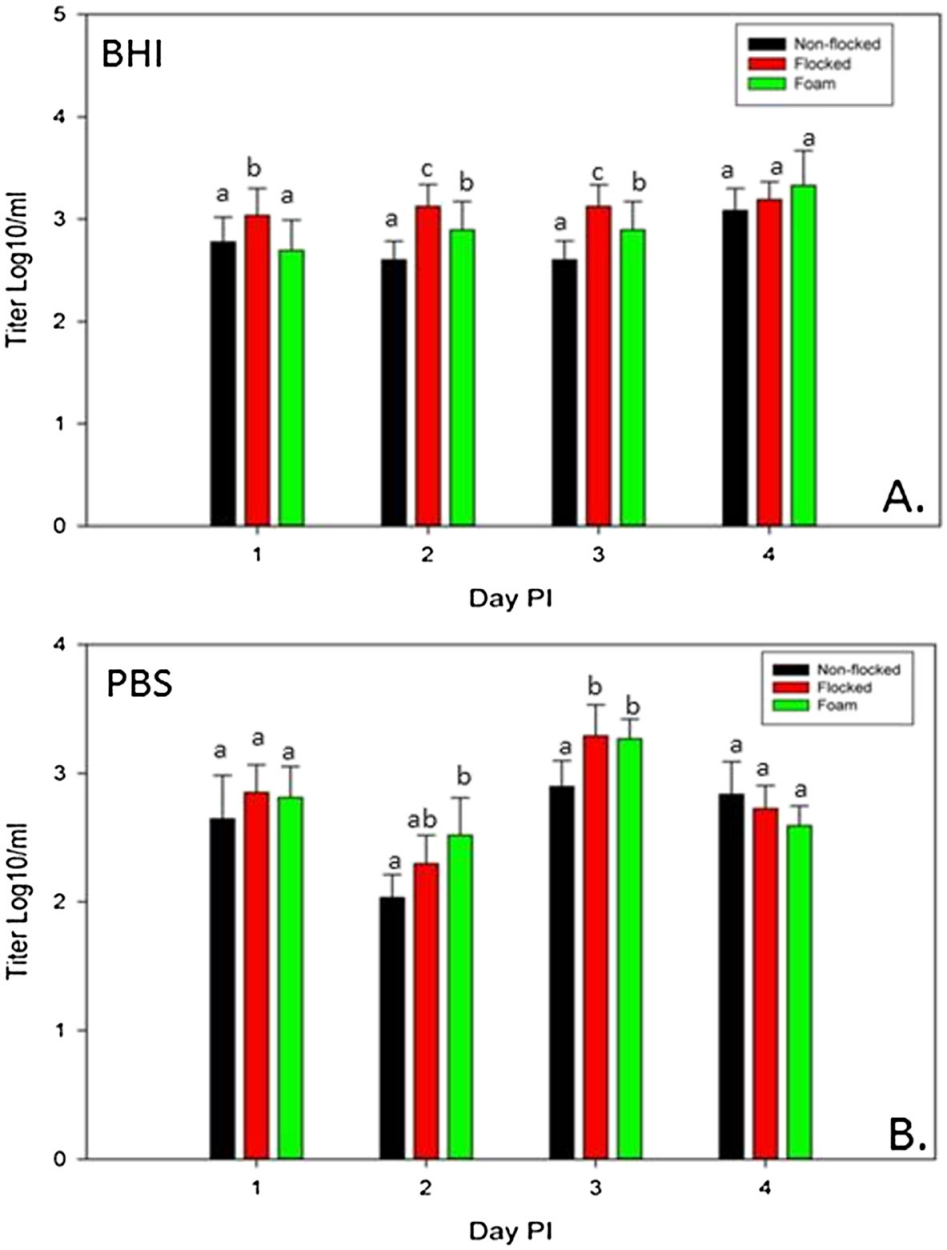
**Mean AIV titers by quantitative real-time RT-PCR for each swab construction type by day post inoculation and transport media: a) brain heart infusion (BHI), b) phosphate buffered saline (PBS).** Statistical significance (p ≤ 0.05) in differences among the amount of virus recovered by swab construction type for each day post inoculation is indicated by different letters above the bars. Error bars represent standard deviation.

All rRT-PCR samples were positive from days 1–4 PI (Table 1). Therefore the titers of virus in the specimens were compared. Too few samples were positive at 7 days PI (DPI) (one foam swab collected in BHI) to make conclusions and all samples were negative for virus detection 10 through 21 DPI. There was significantly more virus recovered (up to half a log_10_) by flocked or foam swabs at 1, 2 and 3 DPI when BHI was used as the transport media and at days 2 and 3 PI when PBS was used as the transport media (Figure 1). This indicates that flocked swabs and foam swabs were better able to capture the most sample and release it into the media. There was no difference in virus detection based on transport media with rRT-PCR. The overall trend was that flocked swabs were marginally better than foam, and both were better than non-flocked swabs in the amount of virus they recovered.

**Table 1:** Results of avian influenza virus isolation from oro-pharyngeal swabs by media and swab type

**Swab type**	**2 DPI**		**3 DPI**		**4 DPI**	
	**BHI**	**PBS**	**BHI**	**PBS**	**BHI**	**PBS**
Non-flocked swab	10/10 (100)^*a^	3/10 (30)^b^	10/10 (100)^a^	7/10 (70)^a^	9/10 (90)^a^	0/10 (0)^b^
Flocked swab	10/10 (100)^a^	5/10 (50)^ab^	10/10 (100)^a^	8/10 (80)^a^	10/10 (100)^a^	0/10 (0)^b^
Foam swab	10/10 (100)^a^	6/10 (60)^ab^	10/10 (100)^a^	9/10 (90)^a^	10/10 (100)^a^	0/10 (0)^b^

Statistical groups (within each day post inoculation) are denoted with letters, where the difference between groups which do not share a letter are statistically significant with a p of value < 0.05. Abbreviations: BHI = brain heart infusion broth, DPI = days post inoculation, PBS = phosphate buffered saline.

*. Number positive/total tested (Percent positive).

Differences in the numbers of positive samples by virus isolation (Table 1) were not statistically significant based on swab construction. However, there were fewer VI positive samples when PBS was used as the transport media at all sample days, and the number of positive samples was significantly lower at 2 DPI with non-flocked swabs and at 4 DPI with all 3 swab types.

The number of positive samples detected by the two commercial AgIA licensed in the US was compared among swab construction type and media with complicated results as swab type appeared to be affected by media (Table 2). With AgIA assay A there were significantly (p value ≤ 0.05) more positive samples with foam and flocked swabs versus non-flocked swabs with BHI, and with PBS there were more positive with non-flocked and flocked swabs than foam. With AgIA assay B, when using BHI, there were significantly more positive samples with flocked swabs than with either other type. With PBS, no samples collected with foam swabs were positive which was significantly different from flocked and non-flocked swabs. Overall, samples collected with flocked swabs appeared to be the most consistently positive by both AgIA assays and were not affected by media type, which correlates with the higher titers of virus from swabbings observed with rRT-PCR.

**Table 2:** Results of commercial antigen immunoassays for AIV with oro-pharyngeal swabs by swab type and media

**Swab type**	**Assay A**		**Assay B**	
	**BHI**	**PBS**	**BHI**	**PBS**
Non-flocked swab	1/45 (2.2)^*a^	12/45 (26.6)^b^	6/45 (13.3)^a^	14/45 (31.1)^ab^
Flocked swab	16/45 (35.6) ^b^	12/33 (36.4)^b^	19/45 (42.2)^b^	13/33 (39.4)^b^
Foam swab	18/45 (40)^b^	1/45 (2.2)^a^	8/45 (17.7)^a^	0/45 (0)^c^

Data from days 2, 3 and 4 post inoculation were pooled. Statistical groups (within each assay [A or B]) are denoted with letters, where groups which do not share a letter have a statistically significant difference with a p of value < 0.05. Abbreviations: BHI = brain heart infusion broth, PBS = phosphate buffered saline.

*. Number positive/total tested (Percent positive).

### Transport conditions

**Figure 2:**
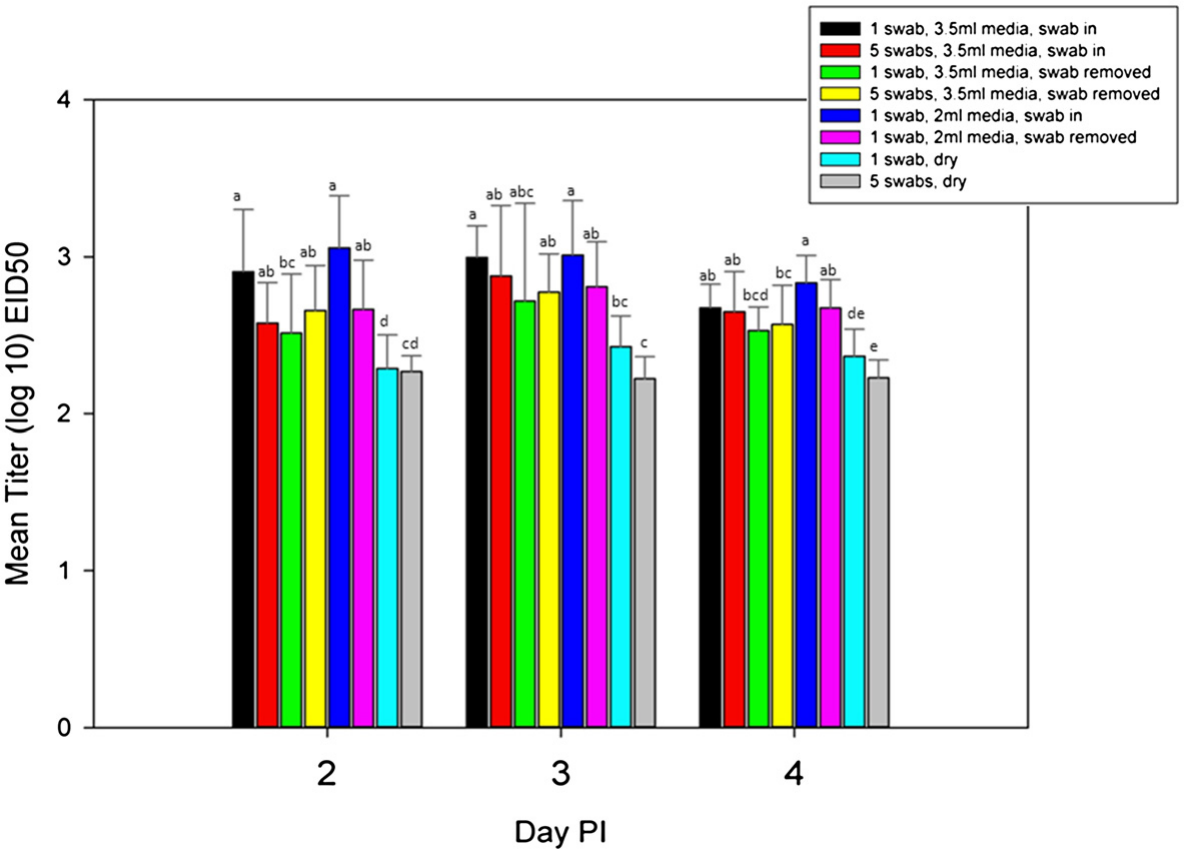
**Mean AIV titers by quantitative real-time RT-PCR for each transport condition.** Statistical significance (p ≤ 0.05) in differences among the amount of virus recovered by transport condition for each day post inoculation is indicated by different letters above the bars. Error bars represent standard deviation.

Virus isolation from swab media (3.5 ml BHI with flocked swabs) did not appear to be affected by whether the swab was left in the vial during transport or removed in the field, nor by the number of swabs (1 or 5) per vial (Table 3). There were fewer AIV positive samples when swabs were transported dry; these differences were statistically significant at day 3 PI with VI. Antigen immunoassays were run with samples from 2, 3 and 4 DPI. There was only one positive sample using assay A which was from the 3.5 ml BHI, swab not removed prior to transport, 5 swab pool group and 2 positive samples with assay B from the same treatment group. Therefore, we were not able to draw conclusions for the AgIA’s, although this does reiterate the lower sensitivity of AgIA’s versus VI and rRT-PCR. By rRT-PCR at 2, 3 and 4 days PI, dry swabs recovered less virus than wet swabs, which was significant for some groups (Figure 2). With rRT-PCR the proportion of positive samples was not significantly different among any of the transport conditions, although there was a clear trend of fewer positives with the dry swabs and rRT-PCR titers were decreased. Also, although not consistently significant, higher titers of virus were detected from groups with a single swab where the swab was left in the vial during transport, suggesting that removing the swab may marginally decrease virus detection.

**Table 3:** Results of testing oro-pharyngeal swabs for avian influenza virus by transport condition

**Transport media**	**Swab removed prior to transport**	**Number of swabs per vial**	**2 DPI**		**3 DPI**		**4 DPI**		**7 DPI**	
			**VI**	**rRT-PCR**	**VI**	**rRT-PCR**	**VI**	**rRT-PCR**	**VI**	**rRT-PCR**
3.5 ml BHI	No	1	10/10 (100)^*a^	15/15 (100)^a^	10/10 (100)^a^	15/15 (100)^a^	10/10 (100)^a^	15/15 (100)^a^	2/10 (20)^a^	2/15 (13.3)^a^
3.5 ml BHI	No	5	10/10 (100)^a^	15/15 (100)^a^	10/10 (100)^a^	15/15 (100)^a^	10/10 (100)^a^	15/15 (100)^a^	2/10 (20)^a^	0/15 (0)^a^
3.5 ml BHI	Yes	1	10/10 (100)^a^	15/15 (100)^a^	9/10 (90)^a^	14/15 (93.3)^a^	10/10 (100)^a^	15/15 (100)^a^	4/10 (40)^a^	0/15 (0)^a^
3.5 ml BHI	Yes	5	10/10 (100)^a^	14/15 (93.3)^a^	10/10 (100)^a^	15/15 (100)^a^	10/10 (100)^a^	15/15 (100)^a^	2/10 (20)^a^	0/15 (0)^a^
2 ml BHI	No	1	10/10 (100)^a^	9/10 (90)^a^	10/10 (100)^a^	10/10 (100)^a^	10/10 (100)^a^	10/10 (100)^a^	0/10 (0)^a^	0/10 (0)^a^
2 ml BHI	Yes	1	10/10 (100)^a^	10/10 (100)^a^	9/10 (90)^a^	9/10 (90)^a^	10/10 (100)^a^	10/10 (100)^a^	0/10 (0)^a^	0/10 (0)^a^
None	No	1	8/10 (80)^a^	12/15 (80)^a^	6/10 (60)^ab^	15/15 (100)^a^	7/10 (70)^a^	14/15 (93.3)^a^	0/10 (0)^a^	0/15 (0)^a^
None	No	5	10/10 (100)^a^	14/15 (93.3)^a^	4/10 (40)^b^	14/15 (93.3)^a^	6/10 (60)^a^	14/15 (93.3)^a^	0/10 (0)^a^	0/15 (0)^a^

Flocked swabs and brain heart infusions broth were used for all sample collection except dry transport where culturettes with self-contained swabs were used. Statistical groups (within each detection test [virus isolation or rRT-PCR] and day post inoculation) are denoted with letters, where groups which do not share a letter have a statistically significant difference with a p of value < 0.05. Abbreviations: BHI = brain heart infusion broth, DPI = days post inoculation, rRT-PCR = real-time RT-PCR, VI = virus isolation.

*. Number positive/total tested (percent positive).

### Media volume

Using BHI and flocked swabs 2 ml of media was compared to the standard of 3.5 ml USDA-APHIS, National Veterinary Services Laboratories, (NVSL). From 2 through 4 days PI 90-100% of samples were positive by both VI and rRT-PCR regardless of media volume (Table 3). At 7 DPI no swabs collected in 2 ml were positive by either VI or rRT-PCR. However, of the swabs collected in 3.5 ml of media, 20% (swab not removed) and 40% (swab removed) were positive by VI and 13.3% (swab not removed) were positive by rRT-PCR (Table 3). So there was a trend toward more positives in the 3.5 ml group at 7 days PI. None of these differences were significant for either VI or rRT-PCR. Additionally, there were no significant differences in titers by rRT-PCR based on media volume alone (Figure 2).

### Swab pooling for AIV detection

**Figure 3:**
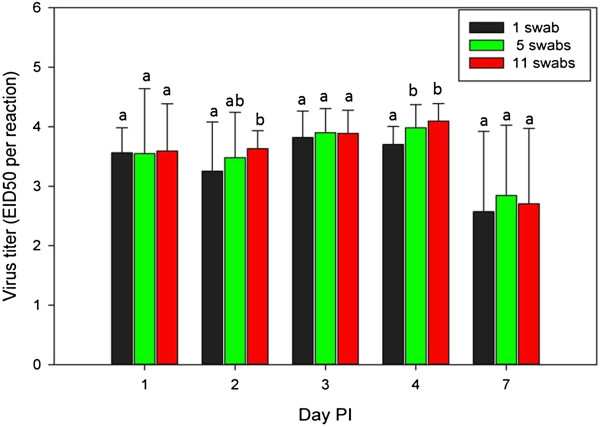
**Mean AIV titers by quantitative real-time RT-PCR for a single swab or pools of 5 or 11 swabs.** Statistical significance (p ≤ 0.05) in differences among the amount of virus recovered by numbers of swabs pooled for each day post inoculation is indicated by different letters above the bars. Error bars represent standard deviation.

Combining 5 or 11 swabs in one tube (1 swab was from a chicken exposed to AIV, the remaining swabs were from uninfected chickens) was compared for AIV detection with VI and rRT-PCR. Single swabs from virus inoculated birds were included as a control. There was no difference in AIV detection from OP swabs among 1, 5 or 11 swabs pooled in a single vial using either rRT-PCR or VI (Table 4). The titer of virus based on rRT-PCR was compared between each swab pool, and on 2 DPI and 4 DPI, single swabs had significantly lower titers of virus as compared to 5 or 11 swab pools (Figure 3).

**Table 4:** Results of testing oro-pharyngeal swabs for avian influenza virus by number of swabs pooled

**Swab pool**	**1 DPI**		**2 DPI**		**3 DPI**		**4 DPI**		**7 DPI**	
	**VI**	**rRT-PCR**	**VI**	**rRT-PCR**	**VI**	**rRT-PCR**	**VI**	**rRT-PCR**	**VI**	**rRT-PCR**
1 swab	10/10 (100)*	20/20 (100)	8/10 (80)	19/20 (95)	10/10 (100)	20/20 (100)	10/10 (100)	20/20 (100)	1/10 (10)	16/20 (80)
5 swabs	10/10 (100)	28/30 (93.3)	10/10 (100)	29/30 (96.6)	10/10 (100)	30/30 (100)	10/10 (100)	30/30 (100)	3/10 (30)	26/30 (86.6)
11 swabs	19/20 (95)	29/30 (96.6)	19/20 (95)	30/30 (100)	20/20 (100)	30/30 (100)	20/20 (100)	30/30 (100)	3/20 (15)	25/30 (83.3)

Non-flocked swabs were collected in brain heart infusion broth and were tested by virus isolation and real-time RT-PCR. There were no statistical differences by number of swabs pooled. Abbreviations: DPI = days post inoculation, rRT-PCR = real-time RT-PCR, VI = virus isolation.

*. Number positive/total tested (percent positive).

### Swab pooling for NDV detection

Similar to AIV, combining 5 or 11 swabs in one vial (1 swab from a chicken exposed to NDV, the remaining swabs from uninfected chickens) was evaluated for virus detection and compared to a single swab. There were no statistical differences or numerical trends in NDV detection based on the numbers of swabs pooled in a vial (Table 5).

**Table 5:** Results of testing oro-pharyngeal swabs for Newcastle disease virus by number of swabs pooled

**Swab source**	**Swab pool**	**1 DPI**		**2 DPI**		**3 DPI**		**4 DPI**		**7 DPI**	
		**VI**	**rRT-PCR**	**VI**	**rRT-PCR**	**VI**	**rRT-PCR**	**VI**	**rRT-PCR**	**VI**	**rRT-PCR**
oro-pharyngeal	1 swab	20/20 (100)	20/20 (100)	20/20 (100)	20/20 (100)	20/20 (100)	20/20 (100)	20/20 (100)	20/20 (100)	1/20 (5)	14/20 (70)
	5 swabs	38/38 (100)	38/38 (100)	38/38 (100)	38/38 (100)	38/38 (100)	38/38 (100)	38/38 (100)	38/38 (100)	1/38 (2.6)	23/38 (60.5)
	11 swabs	38/38 (100)	38/38 (100)	38/38 (100)	38/38 (100)	38/38 (100)	38/38 (100)	38/38 (100)	38/38 (100)	2/38 (5.3)	16/38 (42.1)
cloacal	1 swab	2/20 (10)	7/20 (35)	2/20 (10)	4/20 (20)	12/20 (60)	9/20 (45)	11/20 (55)	13/20 (65)	11/20 (55)	10/20 (50)
	5 swabs	10/38 (26.3)	7/38 (18.4)	3/38 (7.9)	10/38(26.3)	19/38 (50)	28/38 (73.7)	23/38 (60.5)	17/38 (44.7)	15/38 (39.5)	15/38 (39.5)
	11 swabs	8/38 (21.1)	9/38 (23.7)	8/38 (21.1)	11/38 (28.9)	17/38 (44.7)	15/38 (39.5)	27/38 (71.0)	20/38 (52.6)	16/38 (42.1)	17/38 (44.7)

Non-flocked swabs were collected in brain heart infusion broth and were tested by virus isolation and real-time RT-PCR. There were no statistical differences based on the number of swabs pooled. Abbreviations: DPI = days post inoculation, rRT-PCR = real-time RT-PCR, VI = virus isolation.

*. Number positive/total tested (percent positive).

## Discussion

Using swabs collected from chickens experimentally exposed to LPAIV we evaluated numerous elements of sample collection and transport for AIV and evaluated swab pooling with NDV. This data provides concrete information on which factors in sample collection are critical for the most common tests for AIV. Thus allowing surveillance programs to be better tailored to specific situations, including what the best alternatives are when some component of an optimal program is not available.

Based on this data, the optimal method for collecting AIV oral swab samples from poultry would be flocked swabs with BHI media as this provided the best results consistently with all three detection methods. The results with the AgIA were complicated, as there was variation based on assay, swab construction type and media, but the most positives were observed consistently with flocked swabs.

Previous studies [12] have shown the superiority of BHI to PBS for the isolation of AIV, but this had not been evaluated using rRT-PCR, a more commonly used surveillance tool at the present time. The improved capture and release characteristics of flocked swabs have been shown with clinical specimens previously [6,8,9] and one study found that foam swabs were superior to flocked swabs for rapid influenza testing using human specimens [4], which is consistent with our results. Flocked swabs were marginally better than foam, but not always significantly. Therefore, foam swabs could be used if the cost (currently the cost is similar) or availability of flocked swabs prevents their use. Also, while the reasons are not clear at this time, foam swabs should not be used in conjunction with PBS media for AgIA testing since results using both kits were poor. Regarding media, in certain situations where only rRT-PCR will be used and VI will not be attempted (which is rare), PBS which is cheaper and has a longer shelf life at refrigeration temperatures may be used as a transport media.

Transporting the specimen with wet media was most critical for VI, but also seemed to affect detection of AIV by rRT-PCR as titers were reduced. This seemed to contradict a previous study where wet and dry transport of swabs from ducks was compared [3], however the differences in results may be explained by a few methodological differences. The key difference was that virus isolation was not attempted, and this is where the differences were most clear in this study. Furthermore, their samples were from Pekin ducks, which primarily shed cloacally and ours were from chickens, therefore as Roelandt, et a. suggest, the fecal material may protect the virus [3], which we could not evaluate since there were too few positive cloacal swab samples from chickens.

Additionally, it did not matter whether the swab was left in the media during transport or wrung out and removed prior to transport. It should be taken into account that when multiple swabs are left in the media they will absorb it, which reduces the amount available for testing. The advantage of removing the swab prior to transport is that it eases processing for the diagnostic lab, eliminates the loss of media due to absorption by the swabs, and reduces the potential for cross-contamination by aerosols in the event that the swabs are removed in the lab.

Media volume was evaluated because it can have a direct effect on virus concentration and the concentration of inhibitors in a specimen. The initial volume used in a swab tube needs to balance media cost with having adequate final volume to conduct all necessary tests. In addition the initial volume and tube size must allow all swabs to be immersed in the transport media in the event the swabs are left in the tube. The initial volume also needs to account for the number swabs which may be collected in a single vial, since more swabs will absorb more media regardless of how well the material is expressed from the swab. For example a vial in which 11 swabs are collected must have more volume (minimum 5.5 ml) than a vial in which only 5 are collected (minimum of about 3.5 ml). There are two aspects to this question: 1) what should the initial volume be? And 2) if fewer swabs are collected in a vial prepared with a volume for a larger amount will this affect detection? Fortunately initial volume did not have a measureable effect on any of the detection assays with OP swabs.

Swab pooling is used to decrease costs by consolidating the samples from a single premise or flock. Five swabs per vial has frequently been used as a maximum recommended number. An upper limit of 11 swabs has been suggested based on the statistical calculation where 11 swabs from a flock (of 10,000 or more) should be sufficient to detect 25% infection rate with 95% confidence, which is the level of surveillance outlined by the NPIP. A previous study comparing 5 and 11 swabs for detection of LPAIV from broilers by rRT-PCR has been reported and found no difference [2]. Another study comparing a single swab with pools of 5 with samples from turkeys also found no difference [1] This study agrees with those results and adds a comparison with single swabs, five and 11 swab pools in one experiment and very importantly, data for VI. Furthermore, NDV was included in the evaluation of swab pooling because it is an important differential of AIV and pooled specimens are often tested for both AIV and NDV. Swab pooling from NDV infected birds provided similar results, indicating that pooling up to 11 swabs would not affect detection by rRT-PCR or VI.

Although the detection data seems to support that up to 11 swabs can be pooled in a vial, it is critical to note that when implementing testing programs logistics for the field and diagnostic lab must be considered. In this case, a larger vial is needed to assure all swabs are immersed in the transport media if the swabs are going to left in it during transport, and to accommodate an increase in media volume to assure sufficient media is present for both surveillance and confirmation testing which includes virus isolation. The larger 11 swab vial is more difficult to handle, thus increasing the time to process. Also with all the samples in one tube if loss or breakage occurs new samples must be collected. The entire testing process needs to be considered in the cost-benefit assessment of any testing program.

As expected, the differences observed were relatively minor since the detection tests are already so near to their limits of detection, although a few parameters were statistically significant. In addition to providing data on what the optimal methods are, this work also showed that there are some sample collection and transport methods which should be avoided, for example using foam swabs with PBS and transporting swabs dry. BHI was shown to be the optimal vial transport media especially when specimens are collected after 3 days post exposure.

## Conclusions

This work provides practical information which may be applied directly in developing and implementing the optimal surveillance and diagnostic testing programs for AIV and NDV. Flocked swabs performed the best for AIV recovery by both VI and rRT-PCR and were the most consistent with AgIA’s. Foam swabs also performed well with significantly more virus recovery than non-flocked swabs (but significantly less than flocked swabs) based on rRT-PCR at several sample points PI. Brain heart infusion broth was optimal for virus recovery by virus isolation, as there were more positive specimens versus those collected in PBS. Transporting swabs in media appeared to be critical for virus detection by both VI and rRT-PCR, as there were fewer positive with swabs that were transported without media and the mean titers detected by rRT-PCR were decreased. Some of the elements of sample collection and transport that were not observed to impact virus detection were; pooling up to 11 swabs, and whether the swab was removed or left in the vial during transport.

## Methods

### Virus

A low pathogenic H7N2 AIV isolate, A/turkey/VA/SEP-67/2002, from the US Northeast live bird market lineage [13] was selected from the Southeast Poultry Research Laboratory, USDA-ARS (SEPRL) repository because it is a LPAIV isolate which replicates adequately well in chickens to achieve nearly 100% infection. LPAIV was used because it is more difficult to detect due to the relatively mild clinical disease and lower virus shed titers as compared to HPAIV, therefore LPAIV would provide better distinction between methods.

Avian paramyxovirus type-1, also known as NDV was evaluated with swab pooling in addition to AIV. The Roakin strain (mesgonic) was selected from the repository at the USDA-APHIS, National Veterinary Services Laboratories Diagnostic Virology Laboratory, because it would be shed at adequate titers, but without causing mortality.

Both viruses were propagated and titrated by standard methods in embryonated chickens eggs (ECE) [14].

### Exposure of chickens to LPAIV to produce AIV positive swab material

For all experiments, chickens were exposed to the virus and housed in an identical manner. Three to five week old specific pathogen free (SPF) chickens from Southeast Poultry Research Laboratory (SEPRL) in-house flocks were individually tagged and housed in modified Horsfall isolators with *ad libitum* access to feed and water. All chickens were exposed to 10^6.5^ 50% egg infectious doses (EID_50_) of A/turkey/VA/SEP-67/2002 H7N2 LPAIV in 0.1 ml by the intrachoanal route (simulates respiratory/oral transmission). Oro-pharyngeal and CL swabs were collected at all sample times (details of conditions and collection times for each experiment are provided in the description of each experiment below). Chickens which served as a source of negative control swabs were uninfected SPF white leghorn chickens from SEPRL in-house flocks and were housed in battery cages with *ad libitum* access to feed and water. All experiments involving animals were conducted in accordance with the guidelines of the SEPRL institutional animal care and use committee (protocol AUP-FY11-05) and institutional biosecurity committee (protocol IBC-FY12-063-00D).

### Virus isolation

Virus isolation for AIV was attempted with OP and CL swabs in ECE in accordance with standard procedures [14] using three eggs per swab and 200 μl of swab material per egg. Prior to egg inoculation the swabs were treated with antibiotics at a final concentration of: 2 μg/ml amphotericin B; 1,000 Units/ml penicillin G; and 100 μg/ml gentamicin for 1 hour at ambient temperature. Hemagglutination assay using standard methods [15] was used to identify virus replication in allantoic fluid from inoculated eggs. Hemagglutination positive allantoic fluid was also randomly tested with a commercial type A influenza AgIA (Vetscan avian influenza rapid test, Abaxis, Union City, CA) to confirm that the hemagglutinating agent was AIV.

Virus isolation for NDV was conducted in specific pathogen-free ECE with OP and CL swabs according to standard procedures [14] using five eggs per swab pool and 300 μl of swab material per egg. Prior to egg inoculation the swab supernatant was treated with antibiotics at a final concentration of 11,300 Units/ml penicillin G; 2,300 Units/ml streptomycin; 20 Units/ml mycostatin; 750 μg/ml kantrim and 1,150 μg/ml gentomycin.

### RNA extraction and quantitative real-time RT-PCR

RNA was extracted as described by Das et al. [16] using a combination of Trizol LS (Invitrogen, Inc., Carlsbad, CA) and the MagMAX 96 AI/ND Viral RNA isolation kit (Ambion, Inc. Austin, TX) with the KingFisher magnetic particle processor (Thermo Scientific, Waltham, MA). Quantitative rRT-PCR which targets the influenza M gene [17] was performed using the 7500 FAST Real-time PCR System (Applied Biosystems, Foster City, CA), and the AgPath-ID OneStep RT-PCR kit (Ambion, Inc.) in accordance with the NVSL protocols AVSOP1510/001 and AVSOP1521/003. A standard curve for virus quantification was established with RNA extracted from dilutions of the same titrated stock of each virus used to inoculate the chickens and was run in triplicate.

RNA was extracted from NDV specimens with the Ambion MagMAX 96 AI/ND Viral RNA isolation kit with the KingFisher magnetic particle processor in accordance with the manufacturer’s instructions. Real-time RT-PCR for the NDV M gene was conducted as described by Wise [18] on the 7500 FAST Real-time PCR System in accordance with NVSL SOP-AV-1505/0002 and SOP-AV-1521/0003.

### Antigen immunoassay kits

Two commercial lateral flow AIV (type A influenza) AgIA assays: Vetscan avian influenza rapid test, Abaxis, Union City, CA) (assay A) and Flu Detect (Synbiotics, Inc., San Diego CA) (assay B), were used to evaluate OP swabs from 2–4 days PI. Each kit was run in accordance with the manufacturer’s instructions.

### Swab construction type and media comparison

**Figure 4:**
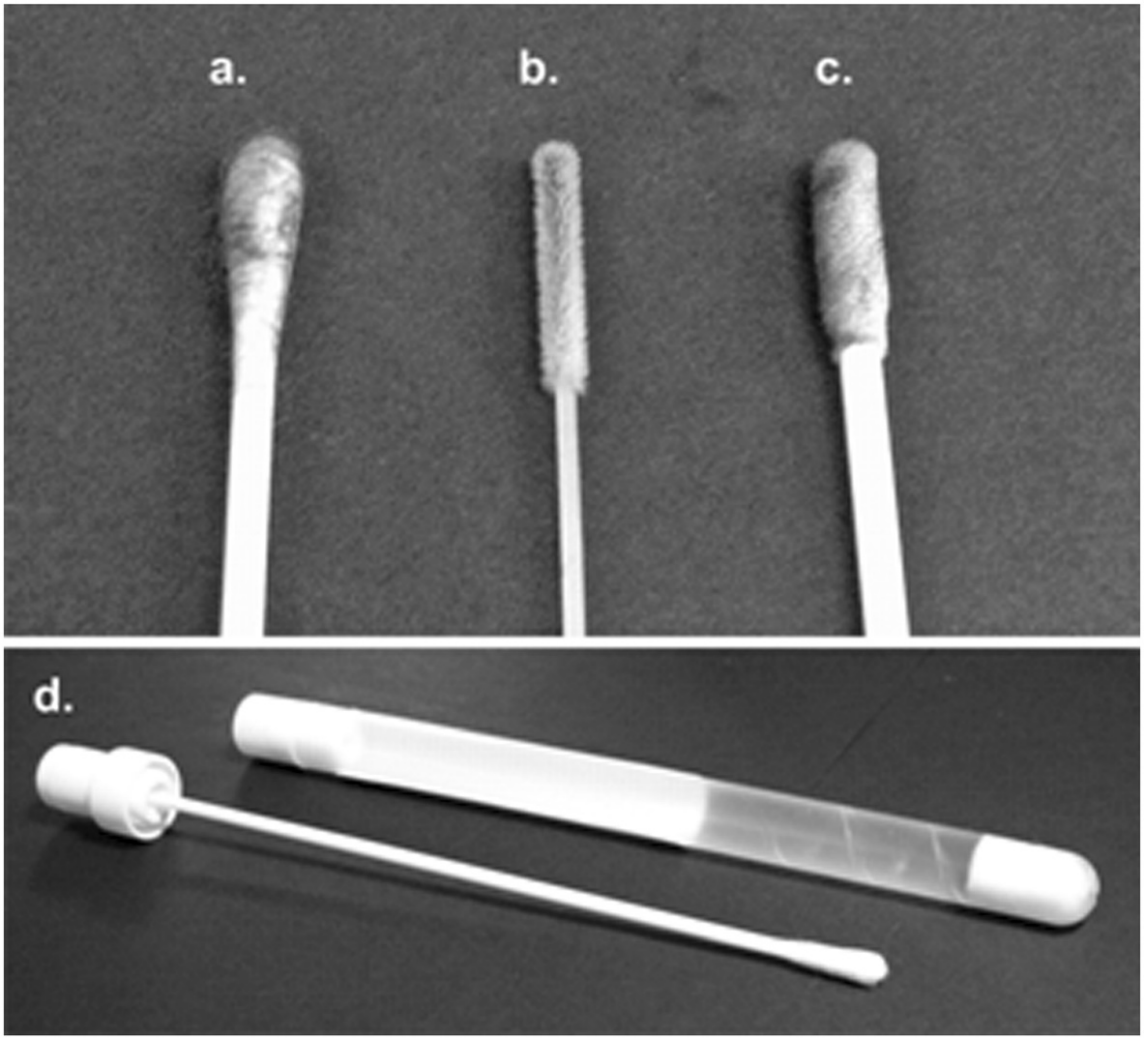
Swab types; a) non-flocked Dacron, b) nylon flocked, c) urethane foam, d) culturette.

**Figure 5:**
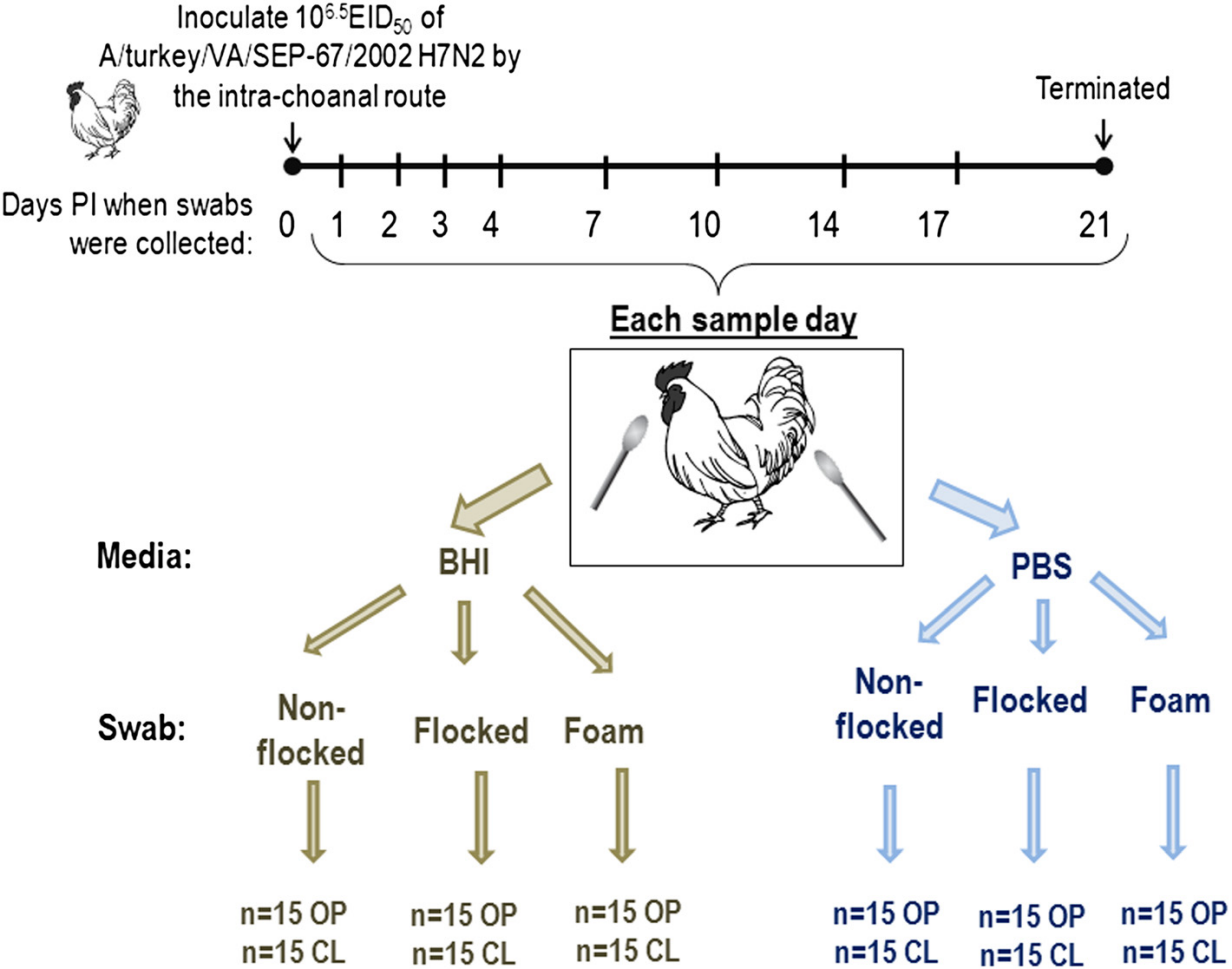
**Diagram of experiment to evaluate swab type and media type.** Specific pathogen free white leghorn chickens were inoculated with 10^6.5^ 50% egg infectious doses of AIV by in the intrachoanal route. Fifteen oro-pharyngeal and 15 cloacal swabs were collected at 1, 2, 3, 4, 7, 10, 14, 17 and 21 days post inoculation using non-flocked swabs, flocked swabs and foam swabs. Individual swabs for each swab construction type were transported in either brain heart infusion (BHI) broth or phosphate buffered saline (PBS).

Three swab types (Figure 4) were evaluated: 1) non-flocked Dacron (wound, polyester) (Fisher Scientific 14-959-97B, Fisher Scientific, Fair Lawn, NJ); 2) nylon flocked swabs (Puritan 3316PN, Puritan Medical, Guilford, ME); 3) urethane foam (Fisher Scientific 140960-3 J). All have plastic shafts, and all have approximately the same diameter head, and cost the same per swab. Each swab type was used to collect OP and CL swabs from 15 birds exposed to LPAIV as described above at 1, 2, 3, 4, 7, 10, 14, 17 and 21 days post inoculation (PI) (Figure 5). Separate groups of birds were used for each swab construction type; each bird was only swabbed once per day.

The swab type evaluation was conducted in conjunction with the media comparison because it was not known if the media could affect sample capture and release from any given swab type. Each of the 3 swab types was collected in both BHI (pH7.2) (Becton-Dickinson and Co., Sparks, MD) and PBS (pH =7.2) (Fisher Scientific). Commercially available powdered concentrates were prepared and sterilized immediately prior to use. Oro-pharyngeal and CL swabs of all three construction types described above were used with each medium (3.5 ml per vial) separately and were collected at 1, 2, 3, 4, 7, 10, 14, 17 and 21 days PI (Figure 5) with the swabs. Separate groups of birds were used for each media type. Individual birds were only swabbed once per day.

### Swab transport conditions

**Figure 6:**
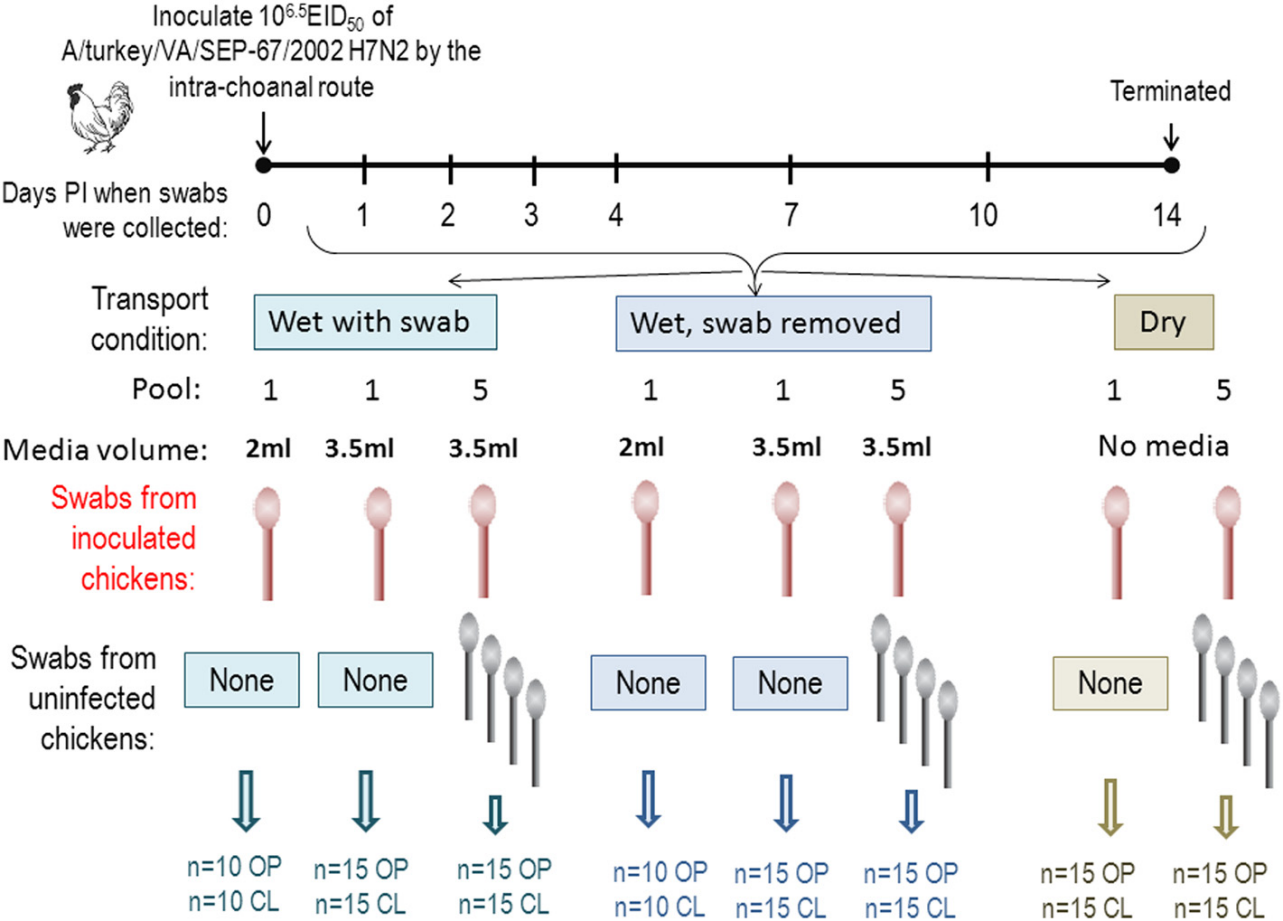
**Diagram of experiment to evaluate transport conditions and media volume.** Specific pathogen free white leghorn chickens were inoculated with 10^6.5^ 50% egg infectious doses of AIV by in the intrachoanal route. Oro-pharyngeal (OP) and cloacal (CL) swabs were collected at 1, 2, 3, 4, 7, 10 and 14 days post inoculation using flocked swabs and brain heart infusion broth (BHI). Individual swabs and pools of 5 swabs (4 from unexposed chickens and 1 from an inoculated chicken) were collected and trans ported in different conditions as follows: 1) collected in 3.5 ml of BHI and transported either in the swab tube n = 15 OP and 15 CL swabs; 2) collected in 3.5 ml BHI and wrung out in the swab tube and removed prior to transport; 3) collected in 2 ml BHI and wrung out in the swab tube and removed prior to transport, n = 10 OP and CL swabs each (individual swabs only); 4) collected in 2 ml of BHI and transported in the swab tube, n = 10 OP and CL swabs each (only individual swabs collected); 5) collected and transported dry, pooled and transferred to BHI 24 hours after transport, n = 15 OP and 15 CL swabs each.

Swabs (flocked swabs) were collected in 3.5 ml BHI media as either a single swab from an infected bird or as a pool of 5 swabs (4 swabs from uninfected birds and 1 swab from an infected bird) at 1, 2, 3, 4, 7, 10 and 14 days PI (Figure 6). Three different swab transport conditions were evaluated: 1) wet swab with the swab left in the vial; 2) wet swab with the swab removed from the vial prior to transport, and 3) dry swab collected with a culturette (Fisherbrand 14-907-20) (Figure 4). Dry swabs were stored for approximately 24 hours at 4°C after collection then placed in 3.5 ml BHI to simulate sub-optimal, but realistic, transport conditions where the swabs were collected one day and not processed until the next. Fifteen swabs were collected at each sample time for each transport condition. The cold chain (4-8°C) was maintained for all sampling conditions.

### Media volume

In conjunction with the “swab transport conditions” experiment described above, a second lower volume (2 ml) set of single swabs were collected to compare the effect of media volume on detection with swabs removed in the field versus swabs left in during transport (Figure 6). Ten swabs were collected at each sample time for both conditions.

### Swab pooling for AIV detection

**Figure 7:**
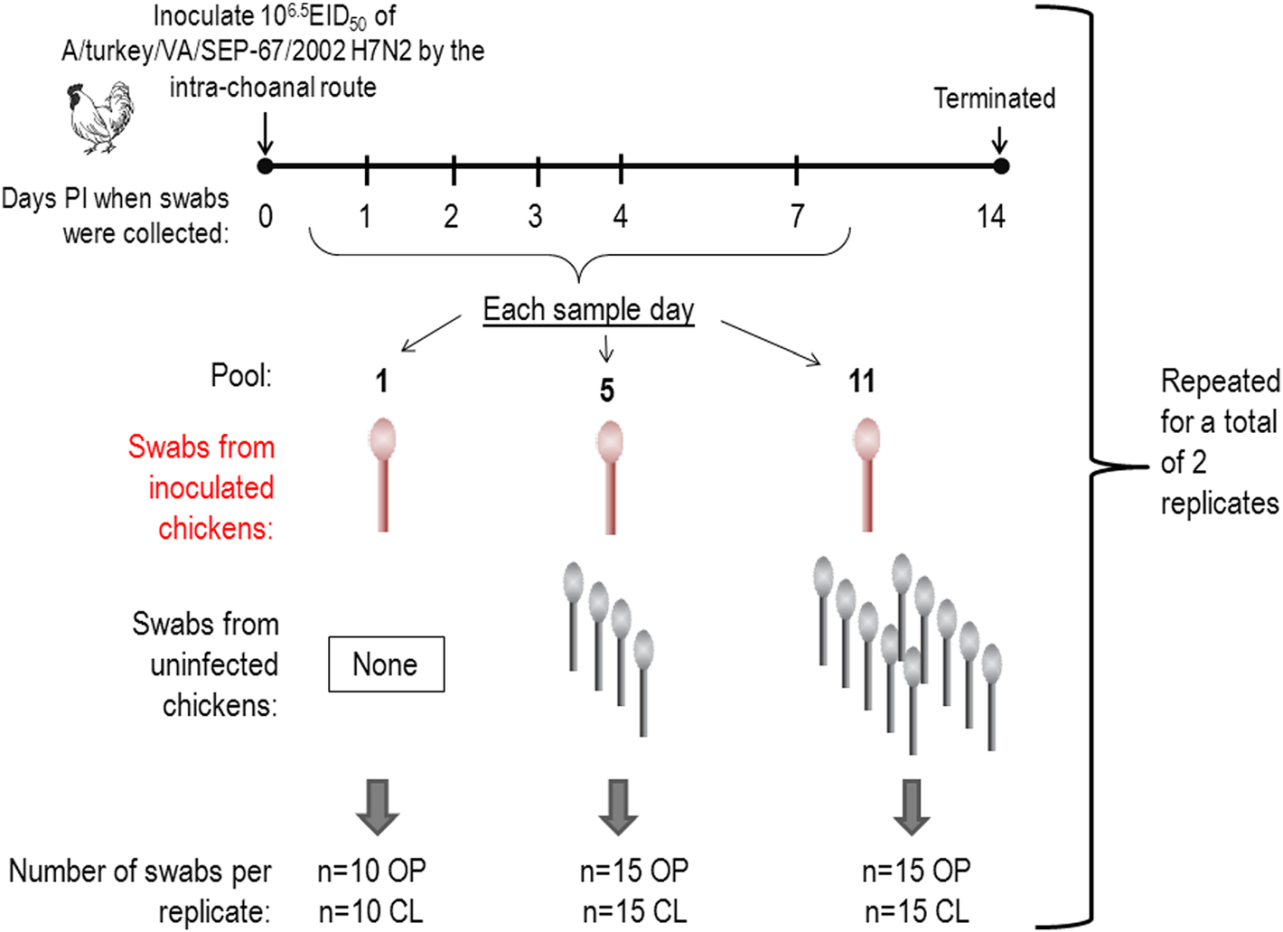
**Diagram of experiment to evaluate swab pooling.** Specific pathogen free white leghorn chickens were inoculated with 10^6.5^ 50% egg infectious doses by in the intrachoanal route. Oro-pharyngeal (OP) and cloacal (CL) swabs were collected at 1, 2, 3, 4, 7 and 14 days post inoculation using non-flocked swabs and brain heart infusion broth. Twenty (2 replicates of 10) individual OP or CL swabs were collected at each time point. Thirty OP and 30 CL (2 replicates of 15) of each of the 5 and 11 swab pools were collected at each time point. Pools were comprised of 1 swab from a chicken inoculated with LPAIV and the remaining (4 or 10) swabs were collected from unexposed chickens.

Pools of varying numbers of swabs were evaluated: a single swab (from an inoculated bird), 5 swabs (1 from an inoculated bird, 4 from unexposed birds), and 11 swabs (1 from an inoculated bird and 10 from unexposed birds). The approach of collecting a single swab from an inoculated bird and the rest from unexposed birds for each pool was used to simulate the diagnostically worst case scenario where only minimal virus is present in the sample. The experiment was conducted so that for each time point there were 15 individual pools of 5 and 11 swabs and 10 single swabs (Figure 7). Swabs were collected at 1, 2, 3, 4 and 7 days PI. Two identical replicates of the experiments were conducted for a total of 20 single swabs, 30 pools of 5, and 30 pools of 11 for each day PI for both OP and CL. Non-flocked swabs were collected in 3.5 ml BHI since the results of the previous experiments showing the better performance of flocked and foam swabs were not available when this experiment was conducted, therefore non-flocked swabs were utilized since these were the recommended swab type used for influenza sample collection at the time.

### Swab pooling for NDV detection

An identical experiment was conducted to evaluate swab pooling with NDV, The experiment was conducted so that there were 19 pools of 5 and 11 swabs and 10 single swabs. Swabs were collected at 1, 2, 3, 4, and 7 days PI. Two identical replicates of the experiments were conducted for a total of 20 single swabs, 38 pools of 5 and 38 pools of 11 for each day for both OP and CL. Chickens were inoculated with 10^6^ EID_50_ of the Roakin strain of APMV-1 (mesogen) by the intra-nasal and oral routes.

### Statistical methods

Within an experiment quantitative rRT-PCR titers among treatment groups were tested for statistical significance with one-way ANOVA within a day PI. If normality failed then Kruskal-Wallis one-way ANOVA on ranks, Dunn’s method was used (SigmaPlot 12.0, Systat Software, Richmond, CA). The proportion of positives between treatment groups within an experiment was tested by Fisher’s Exact test for statistical significance for each assay (VI or rRT-PCR). A p value of ≤ 0.05 was considered to be significant.

## Abbreviations

AgIA: Antigen immunoassay; AIV: Avian influenza virus; APMV-1: Avian paramyxovirus type 1; BHI: Brain heart infusion broth; CL: Cloacal; DPI: Day post inoculation; ECE: Embryonating chicken egg; EID: Embryo infectious dose; HP: High pathogenicity; LP: Low pathogenicity; NDV: Newcastle disease virus; NPIP: National poultry improvement plan; NVSL: National veterinary services laboratories; OIE: World organization for animal health; OP: Oro-pharyngeal; PBS: Phosphate buffered saline; rRT-PCR: Real-time reverse transcription polymerase chain reaction; SEPRL: Southeast poultry research laboratory; SPF: Specific pathogen free; VI: Virus isolation.

## Competing interests

The authors declare that they have no competing interests.

## Authors’ contributions

ES designed and conducted the AIV studies and served as the primary writer of the manuscript; ETM helped conduct the experiments with AIV; JCP designed and conducted the NDV experiments; JG contributed to experimental design of AIV experiments. All authors contributed to data analysis and writing the manuscript. All authors read and approved the final manuscript.
